# The novel mouse mutant, *chuzhoi*, has disruption of Ptk7 protein and exhibits defects in neural tube, heart and lung development and abnormal planar cell polarity in the ear

**DOI:** 10.1186/1471-213X-10-87

**Published:** 2010-08-12

**Authors:** Anju Paudyal, Christine Damrau, Victoria L Patterson, Alexander Ermakov, Caroline Formstone, Zuzanna Lalanne, Sara Wells, Xiaowei Lu, Dominic P Norris, Charlotte H Dean, Deborah J Henderson, Jennifer N Murdoch

**Affiliations:** 1 MRC Harwell, Mammalian Genetics Unit, Harwell, OXON OX11 0RD, UK; 2 Centre for Regenerative Medicine, Chancellor's Building, 49 Little France Crescent, Edinburgh, EH16 4SB, UK; 3 MRC Centre for Developmental Biology, New Hunts House, Kings College, London SE1 1UL, UK; 4 Operations Manager, Research Complex at Harwell, c/o Diamond Light Source Ltd, Diamond House, Harwell Science and Innovation Campus, Oxfordshire, OX11 0DE, UK; 5 MRC Harwell, Mary Lyon Centre, Harwell, OXON OX11 0RD, UK; 6 Department of Cell Biology, University of Virginia Health System, Charlottesville, VA 22908, USA; 7 Institute of Human Genetics, Newcastle University, Newcastle upon Tyne, NE1 3BZ, UK; 8 Centre for Biomedical Sciences, School of Biological Sciences, Royal Holloway University of London, Egham, Surrey, TW20 0EX, UK

## Abstract

**Background:**

The planar cell polarity (PCP) signalling pathway is fundamental to a number of key developmental events, including initiation of neural tube closure. Disruption of the PCP pathway causes the severe neural tube defect of craniorachischisis, in which almost the entire brain and spinal cord fails to close. Identification of mouse mutants with craniorachischisis has proven a powerful way of identifying molecules that are components or regulators of the PCP pathway. In addition, identification of an allelic series of mutants, including hypomorphs and neomorphs in addition to complete nulls, can provide novel genetic tools to help elucidate the function of the PCP proteins.

**Results:**

We report the identification of a new N-ethyl-N-nitrosourea (ENU)-induced mutant with craniorachischisis, which we have named *chuzhoi *(*chz*). We demonstrate that *chuzhoi *mutant embryos fail to undergo initiation of neural tube closure, and have characteristics consistent with defective convergent extension. These characteristics include a broadened midline and reduced rate of increase of their length-to-width ratio. In addition, we demonstrate disruption in the orientation of outer hair cells in the inner ear, and defects in heart and lung development in *chuzhoi *mutants. We demonstrate a genetic interaction between *chuzhoi *mutants and both *Vangl2*^*Lp *^and *Celsr1*^*Crsh *^mutants, strengthening the hypothesis that *chuzhoi *is involved in regulating the PCP pathway. We demonstrate that *chuzhoi *maps to Chromosome 17 and carries a splice site mutation in *Ptk7*. This mutation results in the insertion of three amino acids into the Ptk7 protein and causes disruption of Ptk7 protein expression in *chuzhoi *mutants.

**Conclusions:**

The *chuzhoi *mutant provides an additional genetic resource to help investigate the developmental basis of several congenital abnormalities including neural tube, heart and lung defects and their relationship to disruption of PCP. The *chuzhoi *mutation differentially affects the expression levels of the two Ptk7 protein isoforms and, while some Ptk7 protein can still be detected at the membrane, *chuzhoi *mutants demonstrate a significant reduction in membrane localization of Ptk7 protein. This mutant provides a useful tool to allow future studies aimed at understanding the molecular function of Ptk7.

## Background

Congenital anomalies remain a serious clinical problem, affecting over 15,000 pregnancies per year in the UK, and 110,000 pregnancies per year across Europe [1]. Amongst the most common forms of birth defect are those involving the cardiovascular or nervous systems, in around 30% and 10% of cases, respectively. Basic research into the causes of birth defects is required, in order to enable progress in helping to prevent these disorders.

The mouse is an appropriate model organism for the study of many human birth defects, including nervous system and cardiovascular abnormalities. The developmental basis of mouse neural tube closure and heart formation mirrors that in human gestation, while the ability to genetically manipulate the mouse allows valuable experimental approaches. N-ethyl-N-nitrosourea (ENU) mutagenesis has proven a powerful method for the creation of novel mouse mutants that provide models of human birth defects. Moreover, the random nature of single base substitutions generated by ENU permits the formation of an allelic series of mutations at any locus, facilitating the determination of gene and protein function.

The central nervous system develops from the neural tube, an embryonic precursor that is formed early in human gestation, at around 24-28 days following conception. The morphological events of human neural tube formation are mirrored closely in the mouse, with a well characterised sequence of events that take place between embryonic day (E) 8.5 and 10.5 of mouse development [2]. Neural tube closure initiates at the base of the future hindbrain, termed closure 1, at around the 5 somite stage. Two further sites of closure initiation are observed at about the 12 somite stage, one near the forebrain-midbrain boundary (closure 2) and the other at the rostral extent of the forebrain (closure 3). Continuation of closure between these points results in the completion of cranial closure by around the 17 somite stage, while progressive closure along the spine continues as the embryo elongates, with final closure at the posterior neuropore at about the 30 somite stage.

Failure to complete the normal process of neural tube closure results in neural tube defects (NTDs), which affect around 1 in 1000 established pregnancies [2]. The type of neural tube defect depends on the region of the embryonic axis that is affected. Anencephaly occurs following failure to complete closure in the head, while spina bifida results from disruption of posterior neuropore closure. Failure of the initial event of neural tube closure (closure 1) results in the most severe form of NTD, craniorachischisis, in which almost the entire brain and spinal cord remains open. Anencephaly and craniorachischisis are invariably lethal, owing to the disruption of brain formation. Cases of spina bifida are compatible with postnatal survival but often associated with lower body paralysis or dysfunction. Craniorachischisis accounts for around 10% of NTD cases [3].

Studies with model organisms are essential in helping to elucidate the genetic, molecular and cellular defects that cause these congenital abnormalities which, in turn, provides the potential to identify and develop novel preventative therapies. Over 190 mouse mutants are known with neural tube defects [4] however relatively few of these exhibit the condition of craniorachischisis. Indeed, mutations in only eleven genes leads to craniorachischisis, which makes the identification of novel mutants with this disorder of high scientific and clinical interest.

Strikingly, amongst those mutants with craniorachischisis, almost all genes affect the planar cell polarity (PCP) signalling pathway, either directly or indirectly. The PCP pathway was first identified in *Drosophila*, since disruption of the polarity of cells within an epithelial plane (planar cell polarity) leads to visible phenotypes that include disruption of the regular orientation of hairs on the wings and misorientated ommatidia in the eye. A set of proteins were defined as the core components of the PCP pathway, since they are required for PCP in multiple tissues. These core components include the transmembrane proteins, strabismus/van gogh, flamingo/starry night and frizzled (Fz), and the intracellular protein dishevelled (Dsh). The PCP pathway is also known as the non-canonical Wnt pathway, since it shares components (Dsh and Fz) with the canonical Wnt-β-catenin signalling pathway; however, the *Drosophila *ligand for PCP remains unknown. Several of the mouse craniorachischisis mutants affect genes that are homologues of these core PCP proteins, including *Vangl1 *and *Vangl2 *(strabismus/van gogh homologues), *Celsr1 *(flamingo/starry night), *Frizzled *and *Dishevelled *[5-12]. These genes are required for planar cell polarity in mammals, as evidenced by disruption of the regular orientation of sensory hair cells within the cochlear epithelium in mutants for *Celsr1 *[7], *Vangl2 *[8,10,13], *Vangl1/Vangl2 *[10] and *Fz3/Fz6 *[8], and disruption of the planar arrangement of hair follicles in *Fz6 *[14], *Vangl2 *and *Celsr1 *mutants [15]. Craniorachischisis is also observed following disruption of mouse *Scrib *[16]. Although originally defined as a gene involved in apical-basal polarity rather than planar polarity, several lines of evidence indicate that Scribble affects PCP signalling; mouse *Scrib *mutants demonstrate defects in PCP generation in the inner ear [13], Scrib demonstrates genetic and biochemical interactions with Vangl2 [13,17-19] and *Drosophila *Scrib has recently been shown to play a role in PCP [20]. Similarly, *Ptk7 *mutants exhibit craniorachischisis and PCP defects in the inner ear [21]. *Ptk7 *mutant mice demonstrate a genetic interaction with *Vangl2 *[21], while *Xenopus *Ptk7 forms a complex with Dishevelled and Frizzled7 [22]. Most recently, mutation of *Sec24b *has been shown to cause craniorachischisis, and Sec24b affects PCP through regulation of Vangl2 protein trafficking [23,24].

Here, we report the identification of a new ENU-induced mutant allele that we have named *chuzhoi *(*chz*). Homozygous *chuzhoi *mutants exhibit craniorachischisis, owing to failure to initiate neural tube closure in the future cervical region, closure 1. We demonstrate that the phenotype of *chuzhoi *mutant embryos is consistent with a defect in convergent extension, with a broadened midline and reduced rate of increase in the length-to-width ratio of mutant embryos compared to wild-type. In addition, we demonstrate that *chuzhoi *mutants exhibit disruption of planar cell polarity in the inner ear, and exhibit a genetic interaction with both *Vangl2 *and *Celsr1*. Thus, *chuzhoi *mutants exhibit a number of key characteristics of a planar cell polarity mutant. We show that *chuzhoi *maps to the central region of Chromosome 17, and demonstrate that *chuzhoi *carries a splice site mutation in *Ptk7*. This mutation is predicted to cause the insertion of three extra amino acids into the Ptk7 protein. *Chuzhoi *mutants exhibit disruption of the expression of the Ptk7 protein. This new mutant provides an additional genetic resource to help define the developmental role of the PCP pathway and the molecular function of Ptk7.

## Results

### *Chuzhoi* is a novel mutant with severe neural tube defects

**Figure 1:**
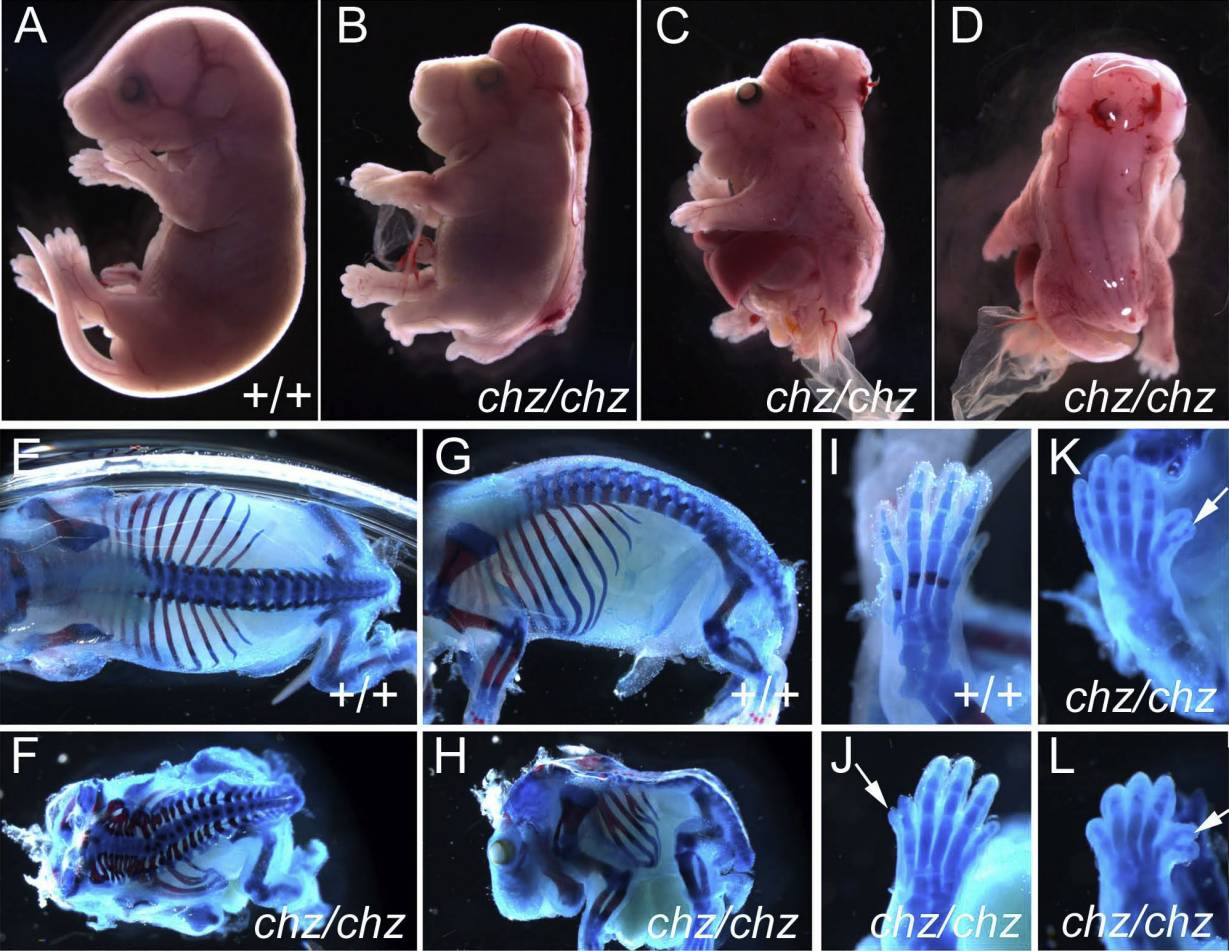
***Chuzhoi *mutants exhibit multiple developmental abnormalities**. (A-D) E17.5 wild-type (A) and *chuzhoi *embryos (B-D) in lateral (A-C) and dorsal view (D). *Chuzhoi *embryos demonstrate the severe neural tube defect of craniorachischisis, where the neural tube is open from the midbrain/hindbrain boundary throughout the hindbrain and spinal cord, and the neural tissue is splayed on either side of the dorsal midline (D). Some *chuzhoi *mutants demonstrate failure to form closed eyelids (C) and a defect in ventral body wall development (likely omphalocele) with protrusion of the liver and guts (C). (E-L) Skeletal preparations of E17.5 wild-type (E, G, I) and *chuzhoi *mutant (F, H, J-L) fetuses stained with alcian blue for cartilage and alizarin red for bone. Dorsal views (E, F) demonstrate shortened and skewed body axis in *chuzhoi *mutants, with splayed vertebrae. Lateral views (G, H) reveal abnormal rib morphology, with fusions and bifurcations. *Chuzhoi *limbs sometimes exhibit polydactyly with an extra digit postaxially (J, arrow) or preaxially (K, L arrows).

*Chuzhoi *arose during a screen for recessive ENU-induced mutations that affect the morphology of mid-gestation embryos [25]. The mutant was identified through the presence of the severe neural tube defect of craniorachischisis, in which the neural tube was open from the midbrain/hindbrain boundary throughout the spinal cord (Figure 1B-D, compare to 1A). Mutants exhibited a shortening and kinking of the body axis and some displayed ventral body wall closure defects (probably omphalocele), with protrusion of the guts and liver (Figure 1C, observed in 48% of fetuses at E16.5 or older; n = 13/27). Mutants also commonly displayed failure of eyelid closure (Figure 1C, seen in 52% of fetuses at E16.5 or older; n = 14/27). Skeletal preparations revealed splayed vertebrae in *chuzhoi *homozygotes, a consequence of the open neural tube defect, and clearly demonstrated a shortening and skewing of the spinal cord (Figure 1F, compare to 1E). Skeletal preparations also demonstrated fusions and bifurcations of the ribs in *chuzhoi *mutants (Figure 1H, compare to 1G) and occasional (~7% fetuses) postaxial or preaxial polydactyly (Figure 1J-L, compare to 1I).

### Severe neural tube defects in *chuzhoi* are consistent with a defect in convergent extension

**Figure 2:**
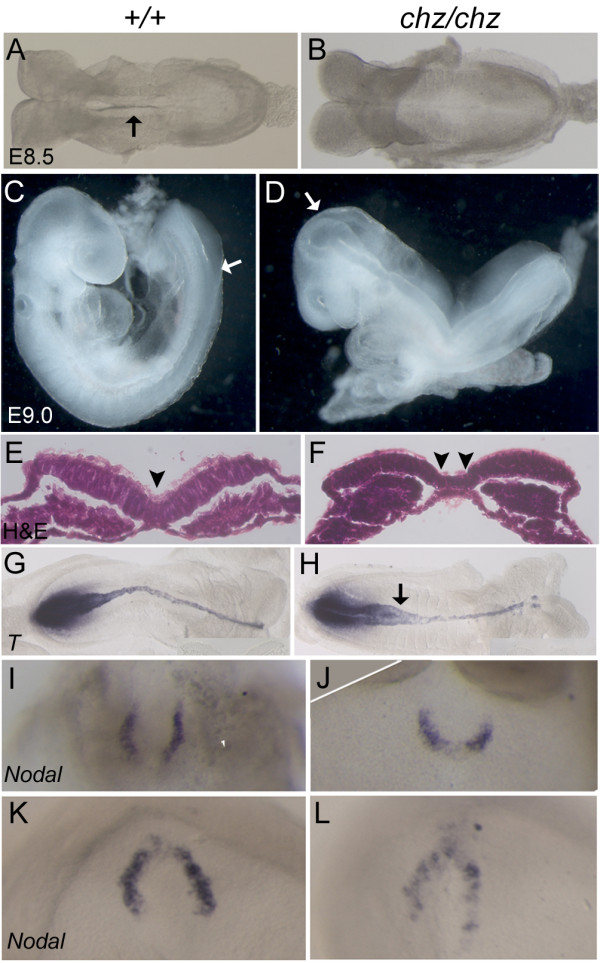
***Chuzhoi *mutants exhibit a failure of closure 1 and a broadened midline**. (A, B) Dorsal views of 6-somite E8.5 wild-type (A) and *chuzhoi *mutant (B) embryos demonstrating initiation of neural tube closure in the wild-type (arrow, A) but failure to initiate neural tube closure in the mutant. (C, D) Lateral views of E9.0 wild-type (C) and *chuzhoi *mutant (D) embryos; arrow marks the most posterior region of closed neural tube. The wild-type embryo has completed closure in the head and only the posterior neuropore remains open in the spine, whereas the *chuzhoi *embryos exhibits closure in the forebrain but remains open from the midbrain along the length of the spinal neural tube. (E, F) Transverse sections stained with haematoxylin and eosin through the caudal end of 5-somite wild-type (E) and *chuzhoi *mutant (F) embryos, revealing a compact ventral midline hinge point in wild-type (arrowhead, E) but a broadened ventral region and split median hinge point in mutants (arrowheads, F). (G, H) Wholemount in situ hybridisation for *brachyury *expression in E8.5 wild-type (G) and *chuzhoi *mutant (H) embryos demonstrates broadened ventral midline in mutants, particularly in posterior regions (arrow, H). (I-L) Wholemount in situ hybridisation for *nodal *expression in E8.0 (head-fold stage) (I, J) and E7.5 (K, L) wild-type (I, K) and *chuzhoi *mutant (J, L) embryos demonstrates shortened and broadened node area in *chuzhoi *mutants at headfold stage (J), while the shape of the node is not obviously altered at E7.5.

Craniorachischisis occurs as a result of failure to initiate neural tube closure. Examination of embryos over the time of closure 1 (E8.5, 4- to 9-somite stage) revealed that in +/+ and *chz/+ *embryos neural tube closure was initiated at the 5- to 7-somite stage and, at E9.0, only the posterior neuropore remained open (Figure 2A,C). In contrast, *chuzhoi *homozygous mutant embryos failed to initiate neural tube closure at E8.5 and, at E9.0, although the forebrain was closed the rest of the neural tube was persistently open (Figure 2B,D), even at later embryonic and fetal stages (Figure 1B-D). Several mouse mutants with craniorachischisis exhibit an abnormal, broadened morphology of the ventral midline of the neural plate at the stage and site of closure 1, as seen in *Vangl2*^*Lp/Lp *^[26], *Scrib*^*Crc/Crc *^[16], *Celsr1*^*Crsh/Crsh *^(unpublished data) mutants and the *Ptk7 *gene trap allele [21]. This defect is thought to mechanically prevent Closure 1 by placing the neural folds too far apart to appose and fuse [26]. We examined *chuzhoi *embryos in order to determine if this morphological defect is present. Histological sections demonstrated a broadened morphology of the neuroepithelium in *chuzhoi *mutants, creating a U-shaped ventral midline that is distinct from the V-shaped midline observed in wild-type embryos (Figure 2E,F). Wholemount in situ hybridization with probes for *Brachyury *and *Shh *confirmed the wider structure of the floor plate and notochord, evident particularly in posterior embryonic regions, just anterior to the primitive streak (Figure 2G,H and data not shown). The morphology of the node appeared abnormal by E8.5 (Figure 2I,J), with a shortened anterior-posterior length and enlarged width in *chuzhoi*, while at E7.5 no reproducible difference in node morphology was apparent between mutant and wild-type embryos (Figure 2K,L).

**Figure 3:**
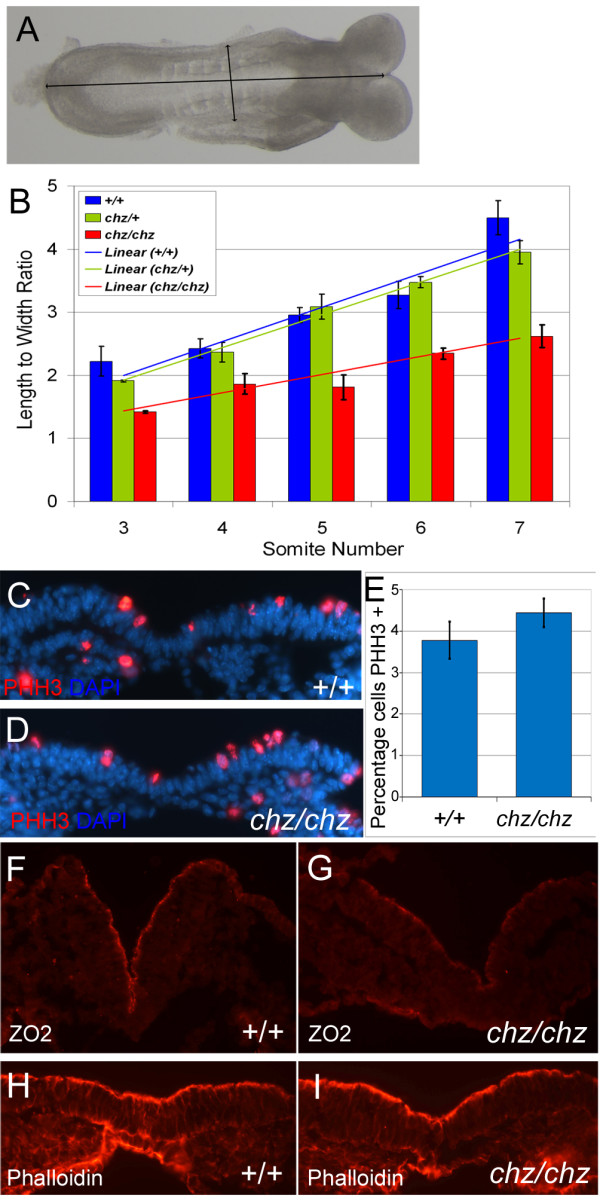
***Chuzhoi *mutants exhibit reduced length to width ratio but no change in proliferation or polarity**. (A) Measurements of midline axial length and width of embryos at the level of the third somite, around the time of Closure 1. (B) Comparison of length to width ratio of wild-type (blue), heterozygous (green) and *chuzhoi *mutant (red) embryos over 3-7 somite stage, demonstrating a reduced rate of increase in the length/width ratio in *chuzhoi *mutants, compared to wild-type and heterozygous littermates. This difference indicates a defect in convergent extension in *chuzhoi *mutants. (C, D) Immunostaining for phosphorylated histone H3 to examine cell proliferation in the neuroepithelium of E8.5 wild-type (C) and *chuzhoi *mutant (D) embryos. Red; phosphorylated histone H3; blue, DAPI. (E) Quantitative analysis of cell proliferation in the neuroepithelium, displayed as the percentage of cells stained for phosphorylated histone H3, revealing no significant difference in proliferation in *chuzhoi *mutant embryos. (F-I) Immunostaining of transverse sections through wild-type (F, H) and *chuzhoi *(G, I) E8.5 embryos with anti-ZO-2 (F, G) and phalloidin (H, I) revealing no substantial difference in expression in *chuzhoi *mutants.

The cause of the broadened midline in *Vangl2*^*Lp/Lp *^mutants has been reported as a defect in the cellular process of convergent extension [27]. A defect in mesodermal convergent extension in the *Ptk7 *gene trap allele has also been reported [28]. To assess convergent extension in *chuzhoi *mutants, we measured the length and width of embryos over the time of neurulation (Figure 3A,B). We found a significant reduction in the rate of increase of the length/width ratio, in *chuzhoi *mutants compared to wild-type and heterozygous littermates (Figure 3B). This is consistent with a disruption of convergent extension in the *chuzhoi *mutants. In contrast, other tissue and cellular characteristics were normal in *chuzhoi *mutants, including rates of cellular proliferation (Figure 3C-E) and apical-basal polarity (Figure 3F-H) within the neuroepithelium at the stage and site of closure 1.

### *Chuzhoi* mutants exhibit disruption of planar cell polarity in the inner ear

**Figure 4:**
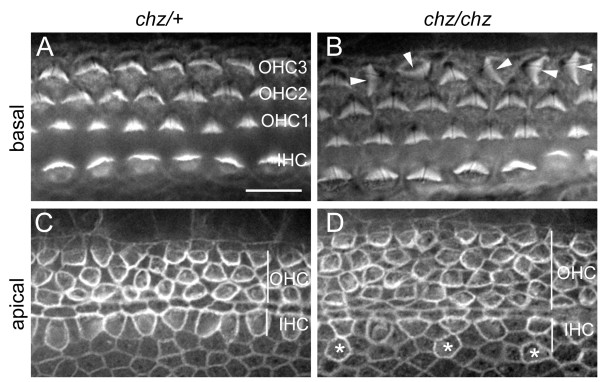
***Chuzhoi *mutants exhibit PCP phenotype in the inner ear**. Phalloidin staining of E18.5 organ of Corti from heterozygous (A, C) and homozygous *chuzhoi *mutants (B, D), with images from the basal (A, B) or apical (C, D) region of the cochlea. Heterozygous mice show an uniformly polarised orientation of stereociliary bundles in the basal region (A), evident on all three rows of outer hair cells (OHC) and the one row of inner hair cells (IHC). Disruption of bundle orientation was most pronounced in OHC3 of the basal cochlea of *chuzhoi *mutants (B), with an apparent randomisation of bundle orientation; arrowheads indicate misoriented bundles. In apical regions, heterozygous mice demonstrate three rows of OHC and one row of IHC (C). In contrast, hair cell arrangement is disrupted in *chuzhoi *mutants (D) with a broadened domain of OHC and four apparent rows of cells as well as extra IHC (asterisks).

Mutants with craniorachischisis often disrupt regulation of planar cell polarity, evident most easily from disruption of the regular orientation of sensory hair cells within the cochlear epithelium. This prompted us to examine hair cell polarity in *chuzhoi *mutants. Phalloidin staining revealed the typical wild-type pattern of three rows of outer hair cells (OHC) and one row of inner hair cells (IHC) with uniformly polarized arrangement of stereociliary bundles in the basal regions of the organ of Corti from E18.5 heterozygous fetuses (Figure 4A). In contrast, basal cochlea regions of *chuzhoi *mutants exhibit disruption of the arrangement of the stereociliary bundles on the third row of outer hair cells (Figure 4B), with randomised cell orientation. Apical regions of the cochlea in heterozygous mice also conform to the normal arrangement of three rows of OHC and one row of IHC (Figure 4C). However, *chuzhoi *homozygous mutants demonstrate a broadened region of OHC with four apparent rows of cells, plus also additional cells external to the row of inner hair cells (Figure 4D). These data reveal that *chuzhoi *mutants have disruption of planar cell polarity in the inner ear.

### *Chuzhoi* mutants exhibit abnormal lung development

**Figure 5:**
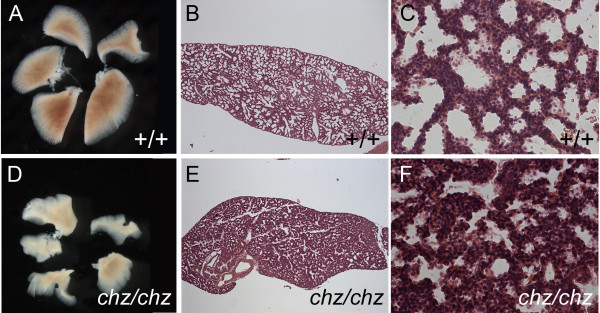
***Chuzhoi *mutants exhibit abnormal lung development**. (A, D) Examination of the gross morphology of the lungs from E18.5 wild-type (A) and *chuzhoi *mutant (D) fetuses showed small and misshapen lobes in the mutants. (B, C, E, F) Haemotoxylin and eosin-stained sections through the left lobe from wild-type (B, C) and *chuzhoi *mutant (E, F) E18.5 fetuses detected thickened interstitial mesenchyme and reduced sepatation in *chuzhoi *mutants.

Our recent observation of lung defects in other PCP mutants [29], prompted us to examine lung morphology in *chuzhoi *mutants. *Chuzhoi *mutants exhibited striking defects in lung development. At E18.5, the lung lobes were reduced in size and highly misshapen (Figure 5D) compared to wild-type littermates (Figure 5A). Histological examination of the lung lobes revealed thickened interstitial mesenchyme with infrequent septation, in homozygous *chuzhoi *mutants compared to wild-type littermates (Figure 5E,F, compare to 5B,C). These defects will result in reduced airway space, and this would be expected to cause breathing difficulties after birth, though this is impossible to determine since mutants die at birth from the severe neural tube defects. Notably, the *chuzhoi *lung phenotype is similar to that observed in other mutants with craniorachischisis, including *Vangl2*^*Lp/Lp*^, *Celsr1*^*Crsh/Crsh *^[29], and *Scrib*^*Crc/Crc *^(Yates et al, in preparation).

### *Chuzhoi* mutants exhibit abnormal heart development

**Figure 6:**
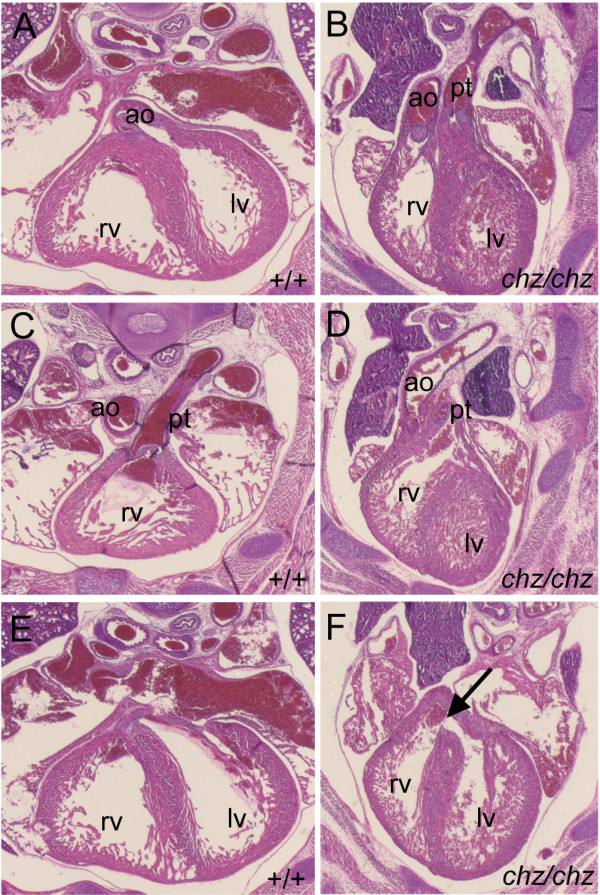
***Chuzhoi *mutants exhibit defects in cardiovascular development**. Transverse sections through the thoracic region of wild-type (A, C, E) and *chuzhoi *homozygous mutant (B, D, F) E17.5 fetuses, stained with haematoxylin and eosin. In control hearts at E17.5, the aorta arises from the left ventricle (A) and the pulmonary trunk arises from the right ventricle (C). In the hearts of *chuzhoi *mutant littermates, the aorta (B) and pulmonary trunk (D) arise from the right ventricle, with the vessels rising parallel to one another (B). Whereas the right and left ventricles are separated in the control heart (E), a peri-membranous ventricular septal defect (arrow in F) can be seen in the *chuzhoi *mutant heart. Ao, aorta; lv, left ventricle; rv, right ventricle; pt, pulmonary trunk.

Several mouse mutants with craniorachischisis also exhibit heart defects, prompting us to examine *chuzhoi *mutants for cardiac malformations. Seven *chuzhoi *homozygous embryos were examined at E17.5 for cardiovascular abnormalities. Of these, five had obvious malformations affecting the outflow region of the heart. In normal hearts, the aorta exits from the left ventricle (Figure 6A) whereas the pulmonary trunk, which gives rise to the pulmonary arteries, exits from the right ventricle (Figure 6C). In contrast, in three *chuzhoi *embryos, the defect of double outlet right ventricle was observed, whereby both the aorta and the pulmonary trunk exit from the right ventricle (Figure 6B,D). This was found, in each case, in association with a ventricular septal defect (Figure 6F, compare to 6E). In the remaining two cases, the connections between the ventricles and the great arteries were concordant, although the vessels were parallel rather than spiralling round one another. Interestingly, in three out of seven hearts examined, the pulmonary arteries, and to a lesser extent the pulmonary veins, were abnormally dilated (data not shown); this did not correlate with the presence of double outlet right ventricle.

**Figure 7:**
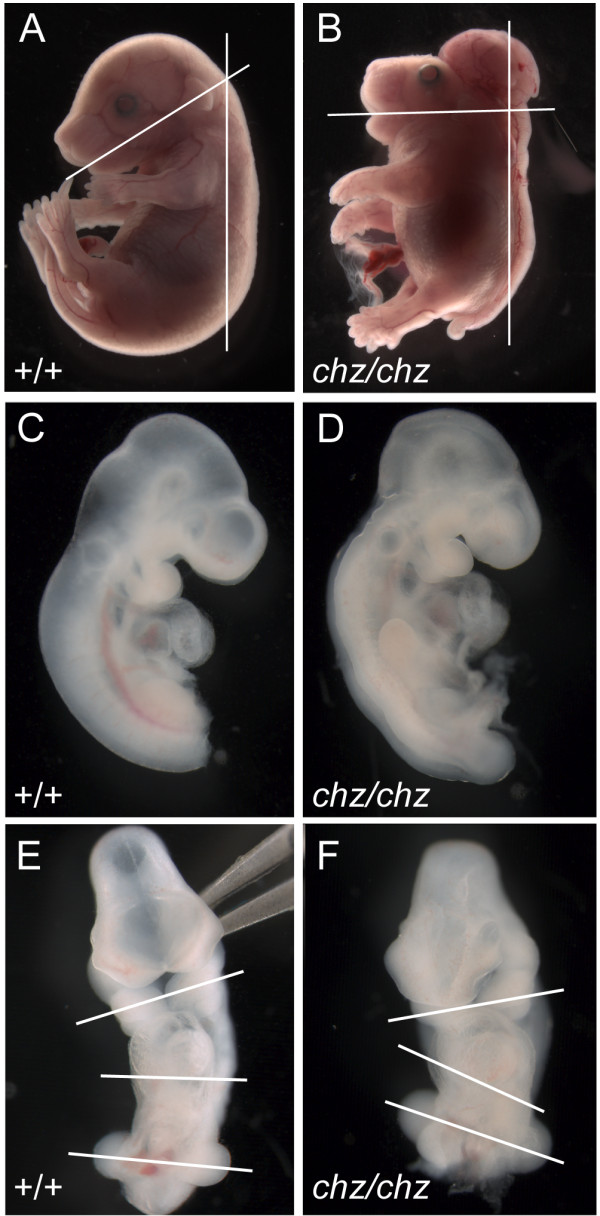
***Chuzhoi *mutants exhibit reduced cervical flexure and abnormal heart looping**. (A, B) Lateral views of E18.5 fetuses demonstrate reduced cervical flexure in *chuzhoi *mutants (B) compared to wild-type littermates (A). Lines are drawn between the mouth opening and centre of the ear, and parallel to the long axis of the spine. (C-F) Lateral (C, D) and ventral (E, F) views of wild-type (C, E) and *chuzhoi *mutant (D, F) E10.5 embryos show abnormal looping of the heart in the mutant embryo. Lines are drawn between the first branchial arches, along the base of the heart, and between the forelimb buds, indicating the abnormal flexure of the mutant embryo; the abnormal positioning of the heart appears to reflect the abnormal skewing of the embryo, likely owing to incomplete embryonic turning. The posterior region of the E10.5 embryos (caudal to the forelimb bud) have been removed to aid photography of the heart region.

The phenotype of double outlet right ventricle in the *Vangl2*^*Lp/Lp *^mutant is attributed, at least in part, to gross abnormalities in embryo morphology, with incomplete embryonic turning and abnormal heart looping. We examined *chuzhoi *mutant embryos for a similar defect. Abnormalities in cervical flexure are observed in homozygous *Vangl2*^*Lp/Lp *^mutants [30]. Similarly, a pronounced reduction in cervical flexure was evident in *chuzhoi *mutant embryos, compared to wild-type littermates (Figure 7A,B). At E10.5, positioning of the developing heart appeared to be abnormal in *chuzhoi *homozygous mutants. The looping of the heart is reduced, but this may be a secondary consequence of the incomplete embryonic turning and consequent abnormal malformation of the embryo; the angle of the malformed heart is similar to the angle of the forelimb buds, in both wild-type and mutant embryos (Figure 7C-F). We propose that the defects in cardiovascular development may, at least in part, be secondary to defects in early heart morphological changes, which may themselves be caused by defects in embryonic turning. The observed kinking of the body axis at later stages, noted above (Figure 1F) is also indicative of incomplete embryonic turning.

### *Chuzhoi* mutants exhibit minor defects in neural crest cell distribution

**Figure 8:**
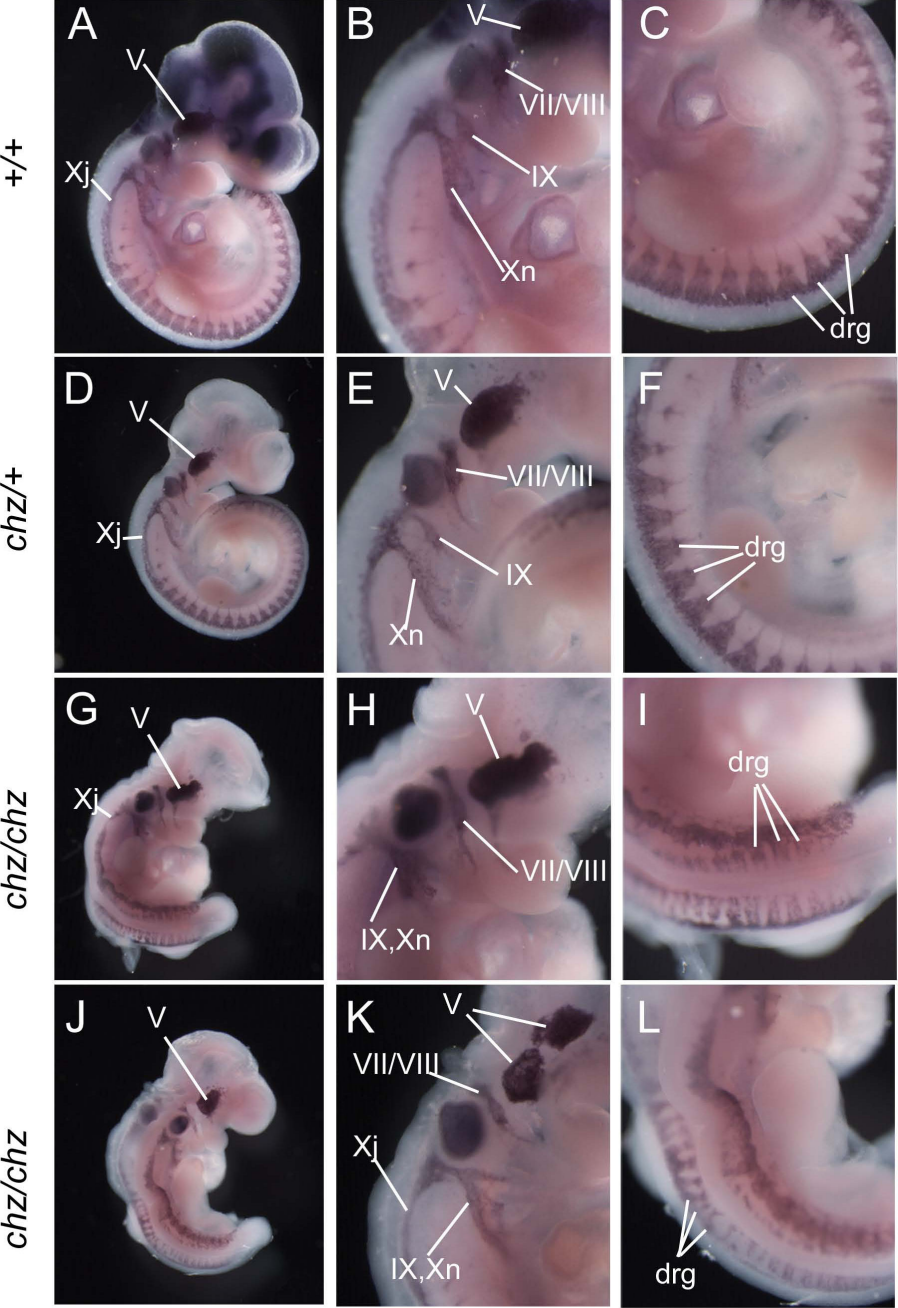
***Chuzhoi *mutants exhibit minor defects in neural crest cell distribution**. (A-L) Wholemount in situ hybridisation for *Sox10 *expression at E10.5 in wild-type (A-C), heterozygous (D-F) and *chuzhoi *homozygous mutant (G-L) embryos. Wild-type and heterozygous embryos showed normal cranial (V, VII/VIII, IX, Xj (jugular) and Xn (nodose)) and dorsal root ganglia (drg). *Chuzhoi *homozygous mutants exhibit abnormally shaped trigeminal ganglia (V), and apparent fusion of the ninth (IX) and tenth (Xn) cranial ganglia. The distrubution of neural crest cells in the spinal region also appears abnormal, with disturbance of the normal V-shaped arrangement of stained cells in the dorsal root ganglia. B, E, H, K are higher power views of the branchial arches (K is left side of embryo shown in J); C, F, I, L are higher power views of the dorsal root ganglia.

Heart defects can arise owing to abnormal neural crest cell specification or migration, prompting us to examine neural crest cell distribution in *chuzhoi *mutant embryos. *Sox10 *is expressed in neural crest cells as they emerge from the dorsal side of the neural tube and in derivatives including cranial and dorsal root ganglia (Figure 8A-F) [31]. Examination of *Sox10 *expression demonstrated the presence of migrating neural crest cells in *chuzhoi *mutant embryos, although their distribution appeared abnormal. In the branchial region, *chuzhoi *mutants exhibited an apparent fusion of the ninth and tenth cranial ganglia (Figure 8G,H,J,K), while the trigeminal ganglion appeared enlarged and misshapen (Figure 8H,K). The distribution of neural crest cells in the spinal region also appeared abnormal, with a narrower domain of *Sox10 *expression and disturbance of the normal V-shaped arrangement of cells (Figure 8I,L, compare to 8C,F). This may be a consequence of the altered morphology of the somites and dorsal root ganglia in the mutant embryos. The anomalies in *Sox10 *expression pattern suggest that although there are some defects in neural crest cell distribution, there is no substantial defect in neural crest cell migration. This conclusion is further supported by several additional observations in *chuzhoi *mutant embryos, including: the presence of approximately normally sized thymic rudiments; comparable *Sox9 *expression patterns in neural crest derivatives; normal α-smooth muscle actin staining in the outflow tract at E11.5, and normal outflow tract septation (data not shown).

### *Chuzhoi* genetically interacts with *Vangl*2^*Lp*^ and *Celsr1*^*Crsh*^ but not *Scrib*^*Crc*^

**Figure 9:**
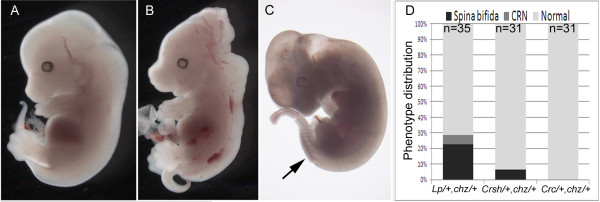
***Chuzhoi *mutants genetically interact with *loop-tail *and *crash *but not *circletail***. (A-C) Intercrosses of *chuzhoi *with *Vangl2*^*Lp/+*^, *Scrib*^*Crc/+ *^and *Celsr1*^*Crsh*^^/+ ^generated doubly heterozygous fetuses with either no overt phenotypic abnormality (A), craniorachischisis (B) or lumbosacral spina bifida (arrow, C). (D) Graphical representation of the proportion of doubly heterozygous embryos demonstrating each phenotype; the number of double mutants examined is given at the top of each column.

The *chuzhoi *mutants exhibit phenotypic similarity with other mouse mutants, including *Vangl2*^*Lp/Lp*^, *Scrib*^*Crc/Crc *^and *Celsr1*^*Crsh/Crsh*^. All four mutants exhibit craniorachischisis and defects in eyelid closure, while a ventral closure defect is observed with high penetrance in *Scrib*^*Crc/Crc *^and lower penetrance in both *Vangl2*^*Lp/Lp *^and *Celsr1*^*Crsh/Crsh *^[7,17,32]. We have previously reported that *Vangl2*^*Lp *^and *Scrib*^*Crc *^genetically interact, such that doubly heterozygous fetuses exhibit craniorachischisis [17]. Similarly, *Vangl2*^*Lp *^and *Celsr1*^*Crsh *^genetically interact, as do *Scrib*^*Crc *^and *Celsr1*^*Crsh*^; in both cases, a proportion of doubly heterozygous embryos demonstrate severe neural tube defects (Damrau et al, in preparation). The *Ptk7 *gene trap allele demonstrates a genetic interaction with *Vangl2*^*Lp *^[21]. These results prompted us to test for a genetic interaction between *chuzhoi *and other mutants with craniorachischisis. Heterozygous *chuzhoi *mice were intercrossed with either *Vangl2*^*Lp/+*^, *Scrib*^*Crc/+ *^or *Celsr1*^*Crsh/+ *^heterozygotes, and embryos examined for a mutant phenotype. In all cases, the proportion of double mutants obtained followed expected Mendelian ratios (Table 1). The phenotypes and penetrance of interactions varied markedly, between mutants. While in all cases, the majority of doubly heterozygous embryos were phenotypically normal (Figure 9A,D), a proportion of *Vangl2*^*Lp/+*^;*chz/+ *embryos exhibited spina bifida (23%) or craniorachischisis (6%, Figure 9B,D), while some doubly heterozygous *Celsr1*^*Crsh/+*^*; chz/+ *embryos displayed spina bifida (6%, Figure 9C,D). In contrast, the doubly heterozygous *Scrib*^*Crc/+ *^*; chz/+ *embryos were all phenotypically normal (Figure 9D). The range of phenotypes, and the penetrance of these defects, is distinct to that observed for intercrosses between other craniorachischisis mutants, such as *Vangl2*^*Lp/+*^*; Scrib*^*Crc/+ *^[17] and *Vangl2*^*Lp/+*^*; Celsr1*^*Crsh/+*^ (unpublished data). The variation of phenotypes and penetrance may be partly attributed to genetic background effects. Nevertheless, the observation of an interaction between *chuzhoi *and both *Vangl2*^*Lp*^and *Celsr1*^*Crsh*^ suggests that the gene disrupted in *chuzhoi* plays a role in modifying PCP signalling in the mammalian embryo.

**Table 1:** Intercrosses between *chuzhoi *and *Vangl2*^*Lp/+*^, *Scrib*^*Crc/+ *^and *Celsr1*^*Crsh*^^/+ ^generate offspring in the expected Mendelian ratios

Cross: *Crc/+ × chz/+*					
Embryo genotypes	*+/+;+/+*	*Crc/+;+/+*	*+/+; chz/+*	*Crc/+;chz/+*	Total

Observed (expected) no. embryos	50 (39.5)	37 (39.5)	40 (39.5)	31 (39.5)	158

Cross: *Crsh/+ × chz/+*					

Embryo genotypes	*+/+;+/+*	*Crsh/+;+/+*	*+/+; chz/+*	*Crsh/+;chz/+*	Total

Observed (expected) no. embryos	44 (38.25)	43 (38.25)	33 (38.25)	33 (38.25)	153

Cross: *Lp/+ × chz/+*					

Embryo genotypes	*+/+;+/+*	*Lp/+;+/+*	*+/+; chz/+*	*Lp/+;chz/+*	Total

Observed (expected) no. embryos	39 (32.75)	30 (32.75)	27 (32.75)	35 (32.75)	131

### *Chuzhoi* carries a splice site mutation in *Ptk7* generating a three amino acid insertion

**Figure 10:**
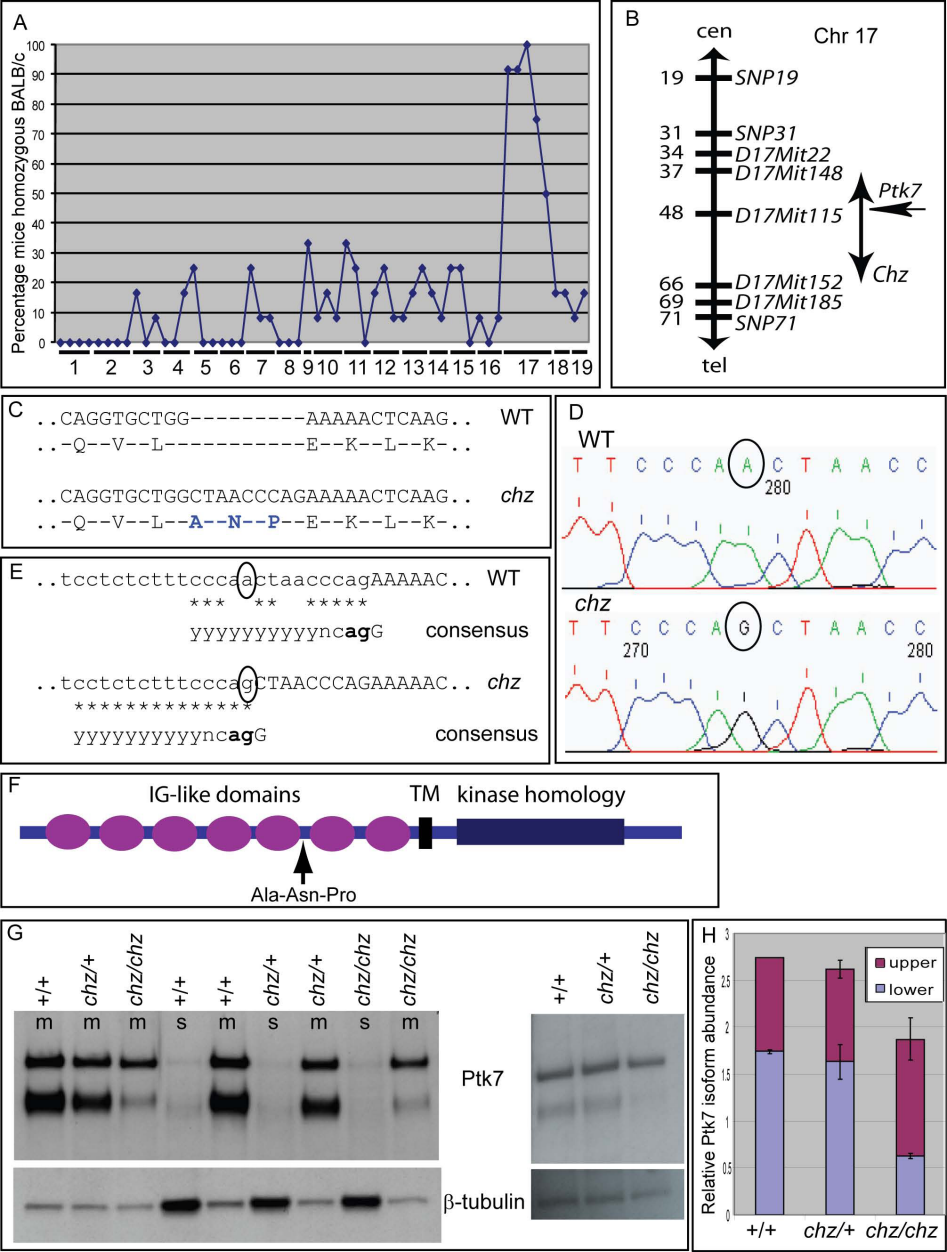
**Positional cloning of *chuzhoi *demonstrates mutation of *Ptk7***. (A) Genome wide scan shows linkage of *chuzhoi *to Chromosome 17; percentage of mice typing as homozygous BALB/c is shown for each marker. (B) Genetic map with additional markers; numbers on left show Mb position along the chromosome, vertical arrow demarcates *chuzhoi *critical region. (C) Sequence and translation of *Ptk7 *cDNA, revealing nine base pair insertion in *chuzhoi*, encoding three additional amino acids (highlighted in blue). (D) Genomic sequence from *Ptk7 *revealing a single nucleotide substitution in *chuzhoi *(circled). (E) Comparison with consensus splice sequence reveals *chuzhoi *mutation (circled) creates a novel splice acceptor site 9 bp upstream to the wild-type site, with greater identity to the consensus sequence (asterisks; identity 14/15 for *chuzhoi*, 10/15 for wild-type). Lower case, intron; upper case, exon; y, pyrimidine; n, any nucleotide; bold, determines intron subtype. (F) Schematic diagram of Ptk7 protein structure with seven immunoglobulin (IG)-like domains, a single transmembrane region (TM) and an intracellular kinase homology domain. The insertion in *chuzhoi *occurs between the fifth and sixth IG domains. (G) Western blot analysis on membrane (m) or soluble (s) protein fractions from E10.5 embryos (left blot), and total cell lysates from E8.5 embryos (right blot) demonstrating changes in Ptk7 protein expression in *chuzhoi *mutants; β-tubulin was used as a loading control. (H) Quantitation of expression of Ptk7 isoforms in wild-type, heterozygous and mutant *chuzhoi *E10.5 embryos, normalised to β-tubulin, and expressed as the abundance relative to that of the wild-type upper isoform.

Genetic mapping using a genome-wide panel of 56 SNP markers on 12 affected individuals revealed linkage to Chromosome 17 (Figure 10A). Analysis with additional microsatellite markers and increased numbers of affected individuals refined the critical region to 28 Mb (Figure 10B). This region contains many genes, including *Ptk7*. Since a gene trap allele of *Ptk7 *exhibits similar phenotypes to *chuzhoi *[21] we examined *Ptk7 *in this mutant. Sequencing of cDNA revealed a nine nucleotide insertion in the *Ptk7 *transcript in *chuzhoi *mutants (Figure 10C) while analysis of *chuzhoi *genomic DNA identified a single nucleotide substitution at the 3' end of *Ptk7 *intron nine (1475-10A > G; Figure 10D). This intron is of the GT-AG subtype, and the mutation creates a splice acceptor site with closer similarity to the consensus sequence [33] than the wild-type (Figure 10E). The new splice acceptor site in *chuzhoi *results in the nine nucleotide insertion in the transcript which encodes three amino acids in the Ptk7 protein (Figure 10C,F). Following breeding of *chuzhoi *to congenicity, all phenotypically abnormal embryos (n = 76, E10.5 or older) genotype as homozygous mutant for *Ptk7*, supporting this mutation as being causative for the craniorachischisis phenotype. Moreover the colony is maintained by genotyping at the *Ptk7 *mutation, with no observed recombination events between this and the *chuzhoi *mutant phenotype. Thus, despite over 200 opportunities for meiotic recombination, *chuzhoi *has not segregated from the *Ptk7 *mutation providing further support to our hypothesis that disruption of *Ptk7 *is causative for the *chuzhoi *mutant phenotype.

Mouse *Ptk7 *encodes a type Ia transmembrane protein of 1062 amino acids with seven extracellular immunoglobulin-like domains and an intracellular kinase homology domain [34]. Analysis by Western blotting with an antibody raised against the extracellular domain of Ptk7 detects two isoforms, of approximately 140 and 100 kDa [21]. Since experimental analyses give no evidence for alternative splicing of mouse *Ptk7 *[34], and there is annotation of only a single transcript in Ensembl (Release 57, March 2010), the 140 kDa band is likely to be full length N-glycosylated Ptk7 protein while the smaller isoform may be a cleavage product [21]. The splicing mutation in *chuzhoi *is predicted to insert the three amino acids alanine-asparagine-proline into the protein, after the fifth IG-like domain (pLeu491_Glu492insAlaAsnPro, Figure 10F). Western blot analysis revealed a change in abundance of Ptk7 in the mutant embryos. In *chuzhoi *embryos at E10.5, abundance of the full length protein was slightly increased but, more significantly, abundance of the smaller isoform was markedly reduced, relative to wild-type littermates (Figure 10G,H) although Ptk7 remains entirely in the membrane fraction of protein extracts. At earlier embryonic stages, around the time of initiation of neural tube closure, the expression of Ptk7 was reduced relative to later embryonic stages and, more notably, the abundance of the smaller isoform was reduced relative to the larger isoform (Figure 10G and data not shown). Nevertheless, the abundance of the smaller isoform was detectably decreased in *chuzhoi *homozygous mutants (Figure 10G). This demonstrates that the insertion in *Ptk7 *in *chuzhoi *mice causes a detectable change in the Ptk7 protein. Together, the mapping, phenotyping, genotyping and protein data provide strong evidence to support our hypothesis that *chuzhoi *is a new allele of *Ptk7*.

### *Ptk7* expression coincides with regions of phenotypic defects in *chuzhoi*

**Figure 11:**
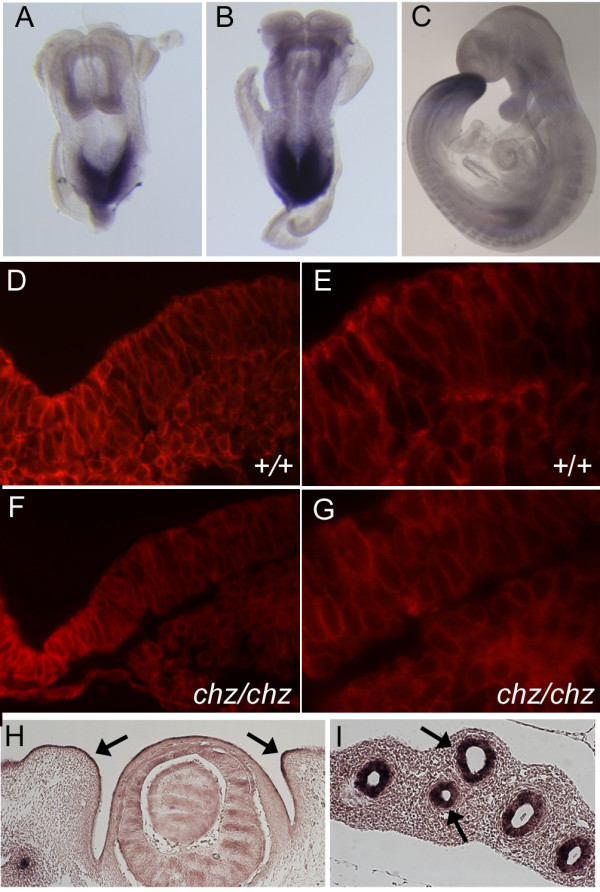
**Ptk7 expression correlates with tissue defects in *chuzhoi***. (A-C) Wholemount in situ hybridisation for *Ptk7 *expression over the time of neurulation reveals robust staining in the caudal region and less intense staining elsewhere; dorsal (A, B) and lateral (C) views of embryos at early somite stage (A), 4 somite stage (B) and E9.5 (C). (D-G) Immunofluorescence with anti-Ptk7 antibody on transverse sections of E8.5 embryos around the site of closure 1. Ptk7 protein is detected in the cell membranes of both mesoderm and neuroepithelium, in both wild-type (D, E) and *chuzhoi *mutants (F, G), although the level of expression is much reduced in *chuzhoi *mutants; exposure times of these images were 57 ms (D) and 192 ms (F). (H, I) Slide in situ hybridisation at E12.5 detects *Ptk7 *transcripts in the developing eye and eyelid epithelium (arrows, H), and intensely in the lung epithelium (arrows, I).

We examined the expression of *Ptk7 *in neurulation stage embryos by wholemount in situ hybridization and immunofluorescence. As reported previously [21,34], *Ptk7 *RNA was first detected at the caudal end of embryos, during head fold and early somite stages (Figure 11A and data not shown). At the 4-somite stage, immediately prior to Closure 1, *Ptk7 *continued to be expressed most intensely at the caudal end of the embryo, while lower levels of expression were detected elsewhere, including the neuroepithelium and somites (Figure 11B). Intense caudal staining continued at later stages, with less intense staining evident elsewhere (Figure 11C). Ptk7 encodes a transmembrane protein, and immunostaining with an anti-Ptk7 antibody detects Ptk7 within the cell membrane [28]. Immunofluoresence on transverse sections of 4-somite stage embryos detected Ptk7 within the cell membranes of both the neuroepithelium and mesenchyme (Figure 11D,E). In contrast, we observed a significant reduction in membrane localization of Ptk7 in the *chuzhoi *mutant (Figure 11F,G, compare to 11D,E and data not shown). It is important to note that in *chuzhoi *mutants, some Ptk7 protein can still localize to the membrane; however, the protein levels are much reduced. The *chuzhoi *mutant images (Figure 11F) required longer exposure times (192 ms) than wild-type sections (Figure 11D, 57 ms) in order to provide broadly comparable photographs, in keeping with the reduced expression detectable by Western blotting. The difference observed in *chuzhoi *mutants adds further support to the argument that *chuzhoi *mutation affects the *Ptk7 *gene.

At later embryonic stages, *Ptk7 *mRNA was detected in several other tissues, including the eyelid epithelium (Figure 11H) and, most intensely, in the lung epithelium (Figure 11I), while sense controls gave no detectable staining (data not shown). Expression of *Ptk7 *in eyelid epithelium correlates with the defect in eyelid closure observed in *chuzhoi *mutants and the *Ptk7 *gene-trapped allele [21]. Notably, several other PCP genes are expressed in the eyelid epithelium, including *Vangl2*, *Scrib *and *Celsr1*, and mutants for these genes also exhibit eyelid closure defects [7,32]. Expression in the lung epithelium is consistent with defects observed in lung development and, again, mirrors the lung expression of *Vangl2*, *Scrib *and *Celsr1*.

### *Chuzhoi *mutants show no alteration in the stability or localization of Vangl2 or Celsr1, and Ptk7 protein shows no disruption in *Vangl2* or *Celsr1* mutants

**Figure 12:**
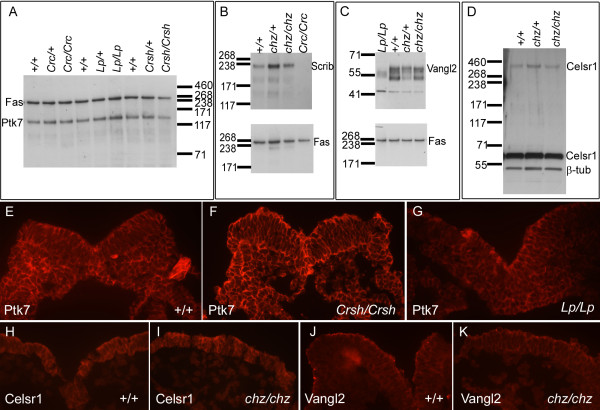
**Examination of expression of Vangl2 and Celsr1 in *chuzhoi *mutants, and Ptk7 in *Vangl2 *and *Celsr1 *mutants**. (A) Western blot analysis of Ptk7 expression in total cell lysates from E8.5 *Scrib*^*Crc/Crc*^, *Vangl2*^*Lp/Lp*^, and *Celsr1*^*Crsh*^^/^^*Crsh *^mutants, compared to heterozygous and wild-type littermates; fatty acid synthase (Fas) was used as a loading control. No obvious difference in Ptk7 expression levels was observed in any of the mutants. (B-D) Western blot analysis of Scrib (B), Vangl2 (C) and Celsr1 (D) expression in total cell lysates from E8.5 *chuzhoi *mutants, compared to heterozygous and wild-type littermates; fatty acid synthase (Fas) or β-tubulin were used as loading controls. Inclusion of protein extracts from *circletail *and *loop-tail *homozygotes were used to help validate anti-Scrib and anti-Vangl2 antibody specificity, respectively. No reproducible difference was observed between *chuzhoi *mutant and wild-type samples, for expression of either Scrib, Vangl2 or Celsr1. (E-K) Immunofluorescence on transverse sections of E8.0 embryos with antibodies for Ptk7 (E-G), Celsr1 (H, I) and Vangl2 (J, K) in wild-type (E, H, J), *Celsr1*^*Crsh*^^/^^*Crsh *^(F), *Vangl2*^*Lp/Lp *^(G) or *chuzhoi *mutant (I, K) embryos. Ptk7, Celsr1 and Vangl2 are detected around the membrane of neuroepithelial cells, and no clear expression difference was observed in the mutant embryos.

We examined the expression of Ptk7 protein in *Vangl2*^*Lp/Lp*^, *Celsr1*^*Crsh/Crsh *^and *Scrib*^*Crc/Crc *^homozygous mutant embryos at the time of onset of neural tube closure, by Western blotting. At this embryonic stage, most Ptk7 protein appeared to be present as the full length form, with barely detectable levels of cleaved isoform. No difference in overall amounts of Ptk7 were observed in any of the mutants, compared to their heterozygous and wild-type littermates (Figure 12A). We examined the cleavage of Ptk7 in *Vangl2*^*Lp/Lp*^, *Celsr1*^*Crsh/Crsh *^and *Scrib*^*Crc/Crc *^mutants, by repeating the Western analysis with E10.5 embryos. No difference in the proportion of full length and cleaved isoforms was observed in any of the mutants, compared to their wild-type littermates, and both isoforms were retained in the membrane fraction in all cases (data not shown). In addition, we examined the expression of Scrib, Vangl2 and Celsr1 in *chuzhoi *mutant embryos, at the time of initiation of neural tube closure. No observable difference was apparent between the amount of Scrib, Vangl2 or Celsr1 protein expressed in *chuzhoi *homozygous embryos, compared to wild-type littermates (Figure 12B-D). Therefore the interaction between *chuzhoi *and *Vangl2*^*Lp *^and *Celsr1*^*Crsh *^appears to be mediated by changes other than expression levels of the proteins.

The genetic interaction may alternatively be mediated through changes in the subcellular localisation of the proteins. We assessed this possibility by immunofluorescence. Ptk7 was detected in the cell membranes of both neuroepithelium and mesoderm in wild-type embryos immediately prior to the onset of neurulation (Figure 12E). A similar distribution of Ptk7 was detected in both *Celsr1*^*Crsh/Crsh *^and *Vangl2*^*Lp/Lp *^mutants at this stage (Figure 12F,G), with no obvious defect in Ptk7 localisation. We also examined the expression of Vangl2 and Celsr1 in *chuzhoi *mutants by immunofluorescence. Celsr1 appears to be restricted to the neuroepithelial cell plasma membrane in wild-type embryos preceding initiation of neural tube closure (Figure 12H), and this distribution was observed in *chuzhoi *homozygous mutant embryos (Figure 12I). Vangl2 protein in wild-type embryos was also localised predominantly in the plasma membrane of the neuroepithelial cells, with an apparent enrichment along apical membranes (Figure 12J). In a number of embryos, we observed an apparent decrease in Vangl2 protein localisation at the apical membrane (Figure 12K), but this difference was not always reproducible and other embryos appeared normal. Scrib localisation appeared similar in wild-type and *chuzhoi *mutants (data not shown). On balance, while other subtle defects may have remained undetected, these data suggest that the interaction between *chuzhoi*, *Crsh *and *Lp *is mediated neither by changes in levels of protein expression nor by alterations in protein localisation.

## Discussion

### *Chuzhoi* is a new mouse mutant revealing further evidence for function of the PCP pathway in neural tube closure

Investigating the function of any gene is greatly helped by the existence of multiple mutant alleles of that gene. ENU treatment of mice has proven to be a powerful method for generating new mutations and, since ENU introduces random, single nucleotide changes, it provides a diverse array of alleles, including nonsense, missense and splice site mutations. These alleles encode a rich resource of genetic defects, often with a spectrum of phenotypic abnormalities that range from complete nulls, through hypomorphs to gain-of-function alleles. An allelic series of genetic mutants is a valuable resource in elucidating the function of the protein.

*Chuzhoi *is a novel mouse mutant that arose from an ENU screen. We have demonstrated that *chuzhoi *maps to the central region of Chromosome 17, and show that *chuzhoi *mutants contain a splice site mutation that results in a three amino acid insertion in the extracellular domain of Ptk7. While we have not fully excluded the possibility that other mutations in the critical interval might contribute to the *chuzhoi *phenotype, we have a number of pieces of evidence to strongly support *Ptk7 *as a very good candidate for the *chuzhoi *mutation. The *chuzhoi *mutant exhibits a number of phenotypes that were previously reported for a gene trap allele of *Ptk7 *[21] including craniorachischisis, abdominal closure defects and eyelid closure defects. In addition, we document abnormal planar cell polarity in the inner ear; both the misorientation of OHC3 cells, and the disrupted arrangement of the rows of OHC and IHC are highly reminiscent of that reported for the *Ptk7 *gene trap allele [21]. Moreover, we have found a disruption in Ptk7 protein expression in the *chuzhoi *mutants.

*Chuzhoi *mutants exhibit the severe neural tube defect of craniorachischisis, owing to failure to initiate neural tube closure. Notably, almost all other mutants with this defect affect genes involved in the PCP pathway. These include those that affect the "core" PCP genes, including *Vangl2, Vangl1/Vangl2*, *Celsr1*, *Frizzled3/Frizzled6*, *Dishevelled1/Dishevelled2 *and *Dishevelled2/Dishevelled3 *[5-12]. Disruption of *Wnt5a *can also lead to craniorachischisis, and *Wnt5a *genetically interacts with *Vangl2 *mutants [35]. Other mutants with craniorachischisis are not "core" PCP genes, but genetically interact with the PCP pathway, such as *Scrib *[16,17]and the *Sfrp1/Sfrp2/Sfrp5 *triple mutants [36,37]. Craniorachischisis occurs following mutation of Sec24b, a protein recently shown to affect membrane localisation of Vangl2 [23,24]. Failure of neural tube closure is seen also in mouse mutants for *Smurf1/Smurf2*, which affect PCP signalling by interacting with phosphorylated dishevelled and Par6, leading to the localised ubiquitination and degradation of prickle [38]. *Zic2/Zic3 *double mutants exhibit craniorachischisis though this may be a result of exencephaly and severe spina bifida, rather than a failure of closure 1; genetic interaction between *Zic *mutants and the PCP pathway has not been assessed [39]. Only a single mouse mutant, *chato *(*Zfp568*), demonstrates failure of closure 1, in a PCP-independent manner [40]. However, the phenotype is more severe than observed in other closure 1 mutants, with very short and broad axes, notochord irregularities distinct from those of the PCP mutants, disruption of heart formation and death at around E9.0 [40].

The phenotype of *chuzhoi *is very similar to that of other PCP mutants and therefore likely indicates a role within the PCP pathway. Indeed, our documentation of polarity defects in the cochlea provide direct evidence that chuzhoi disrupts the PCP signalling pathway. In addition, we have demonstrated a genetic interaction between *chuzhoi *and mutants in two core PCP genes, *Celsr1 *and *Vangl2*. Thus our novel mutant provides additional evidence linking PCP disruption to failure of neural tube closure.

In mutants exhibiting the craniorachischisis phenotype, early embryos display a defect in their morphology, with broadened neural folds, and this defect is thought to preclude neural tube closure by mechanically placing the neural folds too far apart to appose [41]. We show that *chuzhoi *mutants similarly exhibit a broadened midline defect, with a U-shaped rather than V-shaped ventral midline and broadened domains of *Shh *and *Brachyury *expression. The broadened midline defect is thought to arise as a result of disruption of convergent extension, a process first documented in frogs and fish, whereby cell rearrangement and intercalation in the mesoderm and neuroepithelium contributes to narrowing and lengthening of the embryo in a process dependent on the PCP pathway [42,43]. Recent time-lapse live imaging studies have demonstrated convergent extension in mouse embryos, with cell intercalation in the axial mesoderm immediately anterior to the node at E8.5 [44]. Moreover a defect in convergent extension in embryos prior to neurulation has been documented in *Vangl2*^*Lp/Lp *^[27], *Sfrp1/Sfrp2/Sfrp5 *triple [37] and *Par6 *mutants [38], while convergent extension defects of the cochlea are observed in *Vangl2*^*Lp/Lp*^, *Dishevelled1/Dishevelled2 *[45] and *Wnt5a *mutants [35]. The Ptk7 gene trap allele has recently been shown to have defective convergent extension cell movements in the mesoderm [28]. We demonstrate here that the *chuzhoi *mutant embryos have a reduced rate of increase of their length-to-width ratio, compared to wild-type littermates, consistent with disruption of convergent extension.

### Disruption of heart and lung development in *chuzhoi* mutants

The *chuzhoi *mutant exhibits a number of phenotypic similarities to the *Ptk7 *gene trap allele [21] and, in addition, exhibits abnormalities in heart and lung development not reported for the *Ptk7 *gene trap allele. *Chuzhoi *mutants exhibit defects in the heart including double outlet right ventricle (DORV) with a ventricular septal defect (VSD), or parallel arterial trunks. Several other PCP mutants exhibit cardiovascular defects, though the range of defects is only partially overlapping. *Vangl2*^*Lp/Lp *^and *Celsr1*^*Crsh/Crsh *^mutants exhibit DORV and VSD as fully penetrant defects [30,46] while *Scrib*^*Crc/Crc *^mutants exhibit DORV in only a proportion (24%) of embryos examined [47]. DORV is observed in some *dishevelled *mutants, including 38% of *Dvl2-/-*, 36% of *Dvl3-/-*, 38% of *Dvl1-/-;Dvl2-/- *and 32% of *Dlv2+/-;Dvl3+/- *mutants, while others exhibit persistent truncus arterious, in which the outflow tract fails to divide into the aorta and pulmonary artery [9,11]. The common defects suggest the *chuzhoi *mutation may affect PCP-dependent processes in heart development. However, *chuzhoi *mutants also exhibit dilation of the pulmonary artery and pulmonary vein, a phenotype not reported in other PCP mutants, suggesting that Ptk7 has additional, perhaps PCP-independent, roles in development. It is notable that disruption of *off-track *(the chick *Ptk7 *homologue) causes abnormal heart development [48], supporting our hypothesis that the *chuzhoi *phenotype is caused by disruption of *Ptk7*.

The origin of the cardiovascular defects in *chuzhoi *mutants remains to be experimentally determined. Abnormal positioning of the great arteries, including DORV, is thought to result from defects in outflow tract (OFT) rotation during heart development [49]. Evidence for abnormal OFT rotation is supported by the observation of parallel arterial trunks in *chuzhoi *mutants. In *Vangl2*^*Lp/Lp*^, the cardiac alignment defects are attributed in part to abnormalities in cardiac looping [30], as well as defects in myocardialization of the outflow tract cushions [46], which may also affect OFT rotation. Defects in neural crest cell contribution can affect OFT rotation [50] and, indeed, neural crest cells may provide the initiating signal for myocardialization [51]. However, although *Ptk7 *is important for neural crest cell migration in *Xenopus *[22], we found that *chuzhoi *mutants demonstrate only minor abnormalities in neural crest cell distribution in the cranial or spinal regions, and these are likely secondary to the abnormal embryo morphology. In contrast, *chuzhoi *mutants have incomplete embryonic turning, and this defect may cause abnormal looping of the developing heart, with consequential effects on OFT rotation and alignment. Indeed, in chick embryos, siRNA disruption of *off-track *causes failure of bending of the heart loop and abnormal expansion of the ventricular region [48].

### *Chuzhoi* genetically interacts with *Vangl2*^*Lp*^ and *Celsr1*^*Crsh*^ but not with *Scrib*^*Crc*^

The genetic experiments reported here demonstrate an interaction between *chuzhoi *and *Vangl2*^*Lp*^, and between *chuzhoi *and *Celsr1*^*Crsh*^. The difference in the penetrance of phenotypes in the two crosses may be partly attributable to genetic background effects, since *Celsr1*^*Crsh *^is congenic on BALB/c, whereas *Vangl2*^*Lp *^and *chuzhoi *are congenic on C3H/HeH. Background variation may also account, in part, for the different results obtained for the cross between *Ptk7 *gene trap allele and *Vangl2*^*Lp/+ *^reported previously [21] and the cross between *chuzhoi *and *Vangl2*^*Lp/+ *^reported here. The former cross involved a genetic background of approximately 75% C57BL/6, 25% C3H/He in doubly heterozygous individuals and 94% exhibited spina bifida [21]. In contrast, the genetic background of our intercross was almost 100% C3H/HeH. A high proportion of C57BL/6 in the embryos may increase the severity and penetrance of the mutant phenotype; indeed, this is supported by the observation of spina bifida in single *loop-tail *heterozygotes [21]. An increased penetrance of the NTD phenotype on C57BL/6 compared to C3H backgrounds is seen in other mutants, including *vacuolated lens *[52] and *circletail *(our unpublished data).

The evidence of a genetic interaction between *chuzhoi *and both *Vangl2*^*Lp *^and *Celsr1*^*Crsh *^indicate that *chuzhoi *can influence the PCP pathway, although the molecular mechanism of this interaction remains unclear. We have demonstrated that the expression level and membrane localization of Ptk7 are not detectably disrupted by mutation of either Vangl2 or Celsr1 and, similarly, Celsr1 expression and localization appear unaltered in *chuzhoi *mutants. We have some data to suggest that Vangl2 localisation may be subtly altered in *chuzhoi *mutants, with a reduction in protein at the apical membrane. *Xenopus *PTK7 has been shown to localize dishevelled to the plasma membrane, by forming a complex with dishevelled and frizzled7 [22]. It is possible that mammalian Ptk7 might act in the PCP pathway in a similar way, perhaps forming a complex involving Dishevelled, Frizzled, Vangl2, and Celsr1 [15,18,19,53] although detailed biochemical studies are required to confirm this hypothesis. However, disruption of Ptk7 does not alter Dvl2 localisation [28]. Moreover, yeast 2-hybrid experiments with the intracellular domain of mouse Ptk7 as bait against three different libraries isolated neither dishevelled nor frizzled, nor other members of the PCP pathway (data not shown). Further experiments are required to ascertain the precise molecular function of mammalian Ptk7.

We did not detect a genetic interaction between *Scrib*^*Crc *^and *chuzhoi*. While we cannot fully exclude genetic background effects, since both *Scrib*^*Crc *^and *chuzhoi *are congenic on C3H/HeH (similar to *loop-tail*), we believe that genetic background does not play a large contribution to the absence of mutant phenotype in the *Scrib/chuzhoi *double heterozygotes. Rather, we suggest that the absence of an observed genetic interaction between *Scrib*^*Crc *^and *chuzhoi *implies that Scrib and Ptk7 may affect the PCP signalling pathway through distinct mechanisms.

A number of mutants demonstrate a trans-heterozygous interaction with *Vangl2*^*Lp *^mice, and this is commonly used as evidence for a function within the PCP pathway. The *Vangl2*^*Lp *^mutation is semi-dominant, and *Vangl2*^*Lp/+ *^mice exhibit a number of disorders including delayed closure of the neural tube [54]. This delay means that *Vangl2*^*Lp/+ *^embryos are more sensitive than wild-type strains to embryonic conditions, so that sub-optimal environmental or genetic conditions that alone have no observable effect, may cause an NTD phenotype when crossed to *Vangl2*^*Lp/+*^. It is possible, therefore, that apparent genetic interactions can occur as a result of non-specific effects on embryonic conditions, rather than from a molecular pathway intersection. In this regard, it will be important to ascertain additional (molecular) data to confirm interaction specificity.

### Molecular function of Ptk7 protein

Ptk7 is similar to the receptor protein-tyrosine kinase family of proteins, with seven extracellular immunoglobulin-like domains, a single transmembrane motif and an intracellular kinase homology domain. However, the kinase homology domain lacks the DFG triplet that is necessary for the chelation of Mg2+ during phosphotransfer and required for kinase activity [34,55,56]. Ptk7 is therefore thought to encode a pseudokinase, lacking catalytic function. Although other proteins lacking the DFG motif have been found to have kinase activity through the use of an alternative motif [57], experimental evidence confirms that mammalian Ptk7 lacks kinase activity and is not phosphorylated in cell culture [34,55]. Ptk7 orthologues in *Hydra *(Lemon) and chicken (KLG) also lack kinase activity [58,59]. In contrast, the *Drosophila *homologue of Ptk7 (Off-track, Otk, previously named Dtrk), mediates calcium-dependent homophilic binding, and is phosphorylated on tyrosine residues in cultured cells [60]. Ptk7 has been proposed to be part of an unusual receptor tyrosine kinase signalling mechanism involving transmembrane domain-mediated interactions rather than kinase activity [58], or to mediate signal attenuation [59]. The transmembrane domains of the Ptk7 family are unusually highly conserved, across evolution, and this has been argued to support an important role for this domain [61]. Notably, the kinase-like domain of *Xenopus *PTK7 is required for the physical interaction of PTK7 with dishevelled [22].

### Ptk7 protein exists as at least two isoforms in mouse

Western blotting of mouse Ptk7 detects two isoforms, of approximately 100 and 140 kDa, using an anti-Ptk7 rabbit polyclonal antibody raised against the extracellular domain of Ptk7 [21]. Although human PTK7 encodes five alternatively spliced variants [62], there is no evidence for alternative splicing of mouse Ptk7. An alternative hypothesis is that Ptk7 undergoes protein cleavage. Intriguingly, generation of the extracellular domain of human PTK7 as a soluble fusion protein gives rise to a band of approximately 100 kDa [63], similar in size to the lower band observed endogenously and raising the possibility of juxtamembrane cleavage of Ptk7. Juxtamembrane cleavage of other type I transmembrane proteins commonly involves ADAM proteases [64-66], but this ectodomain cleavage event usually results in the production of a soluble factor [67,68]. In contrast, the smaller isoform of Ptk7 remains within the membrane fraction of cells, indicating that this putatively cleaved isoform is not freely soluble but remains either integral to the membrane or closely associated with membrane proteins. Comprehensive biochemical experiments are required to elucidate the precise basis of the two isoforms observed by Western blotting, and to investigate the possible existence of an intracellular fragment following cleavage.

Regardless of the precise mechanism of formation of the lower molecular weight isoform, it is intriguing that *chuzhoi *mutants reveal decreased abundance of this smaller isoform on Western blots. Since the insertional mutation in *chuzhoi *occurs between the fifth and sixth immunoglobulin domains, this cannot directly disrupt a juxtamembrane cleavage site, although it may inhibit cleavage indirectly, for example, by protein misfolding. Alternatively, the *chuzhoi *mutation may result in reduced stability of the smaller isoform. The reduced abundance of the smaller isoform in *chuzhoi *raises the possibility that this may be the biologically active moeity. However, the relative abundance of the smaller isoform is much reduced at earlier embryonic stages, including at the onset of neurulation, arguing against this hypothesis. An alternative suggestion is that the insertion in *chuzhoi *disrupts function of the full length Ptk7 protein through protein misfolding. Further biochemical studies on the protein function may help to distinguish between these possibilities.

## Conclusions

Our results with a new mouse mutant provide additional evidence that disruption of the planar cell polarity signalling pathway causes defects in neural tube, heart and lung development. This mutant provides a new genetic tool to help investigate the function of the PCP pathway and the precise molecular role of the likely candidate gene product, Ptk7 protein.

## Methods

### Mice and embryos

*Chuzhoi *was identified during a three-generation (G3) recessive mutagenesis screen [25], in which BALB/c males were injected with ENU and outcrossed to C3H/HeH. Male F1 offspring were mated to C3H/HeH and F2 females backcrossed to their father. G3 embryos were examined at E13.5 for developmental abnormalities. Affected fetuses were used for genetic mapping with a 55-marker genome-wide SNP panel (sequences available on request), and additional microsatellite markers. The mutant line was maintained by backcrossing to C3H/HeH and, following identification of the mutation in Ptk7, genotyped for this mutation, by pyrosequencing. Mice heterozygous for *Vangl2*^*Lp *^[6] and *Scrib*^*Crc *^[16] mutations were maintained on C3H/HeH, while the *Celsr1*^*Crsh *^mutant [7] was maintained on BALB/c. Animals were maintained following guidelines of the Medical Research Council and in accordance with the Animals (Scientific Procedures) Act, 1986.

Mice were maintained routinely on a 12 h light-dark cycle (dark from 19:00 to 07:00 h). Embryos were generated by overnight matings, with the day of finding a copulation plug designated as embryonic day (E) 0.5. Some mice were maintained on a reverse light-dark cycle (dark from 10:00 to 22:00 h), and litters from these animals were designated as E1.0 on the day of plugging. Embryos were dissected in PBS with 10% newborn calf serum and processed according to downstream application.

Mice and embryos were genotyped by pyrosequencing, either at the mutation itself (for *Chz*, *Lp*, and *Crsh*) or at closely flanking SNPs (for *Crc*). Primer sequences were: Chz_For NNNGGATGGCCCTGCCTCTTTCT, Chz-Rev 5'Biotin-GGAGGTGGCGTGAACTTGAG, Chz-seqF CCTCCTCTCTTTCCCA; Crsh-For NNNGAGAACAGCCCTGTGGGTTCA, Crsh-Rev 5' Biotin-CATTGCCCTCCACGATCTGA, Crsh-seqF GAATAAGGGCCAACG; Crc-74-For NNNCATTGGAAAACATGGGGAGGA, Crc-74-Rev 5'Biotin-AGCATCAGGGACAGGCAAGG, Crc-74-seqF AAAACATGGGGAGGAC; Crc-76-For 5'Biotin-GACAGTGGGCAAGGCTGACA, Crc-76-Rev NNNGGCTGCACTTGTCGCTCAGA, Crc-76-seqR GCTCAGAGGACTCTCATC; Lp-For 5'Biotin-GTCCTGGCGCTTCAAGAGGA, Lp-Rev NNNGGCCAAACAGTGGACCTTGG, Lp-seqR CAGTGGACCTTGGTGA.

### Sequencing

RNA was extracted from E8.5 embryos using GenElute (Sigma) and reverse transcribed with MMLV-RT (Invitrogen). DNA and cDNA were amplified with intron or exon-specific *Ptk7 *primers (sequences available on request), purified using Qiaquick (Qiagen) then sequenced with BigDye reagent (ABI) and ABI3700.

### Histology and in situ hybridisation

Histology and skeletal preperations used standard protocols. Wholemount in situ hybridisation was performed essentially as described [69], using digoxigenin-labelled riboprobes for *Ptk7, Shh, Brachyury, Sox10 *and *Sox9*. To generate a probe for *Ptk7*, an 828 bp fragment corresponding to region 981-1808 bp of the cDNA was amplified using primers F (5'-TGGTGATGAGGAACGAGTCA) and R (5'- GTGGCCCTGGTACACAGTTG) and cloned into pGEM-T (Promega). At least three embryos of each genotype were analysed with each probe and processed under identical conditions. Embryos were photographed on a Leica MZ16 stereomicroscope and vibratome sectioned at 35-50 μm as described [70]. Slide in situ hybridisation was performed using 12 μm wax sections as described elsewhere [6], with digoxigenin-labelled riboprobes for *Ptk7.*

### Measurement of length-width ratios

For length and width measurements, E8.5 embryos (3-7 somites) with embryonic membranes dissected off were flat-mounted on a microscope slide in a drop of PBS/10% fetal calf serum. Embryos were flattened slightly under a coverslip, supported with dabs of silicon grease to prevent the embryos from being squashed. Measurements were taken as the length of the embryo along the axial midline, from the boundary with the allantois posteriorly to the ventral midline of the open cranial neural folds anteriorly (Figure 3A). The width of the embryo was measured as the extent of the lateral plate mesoderm, at the level of the third somite in each case (Figure 3A).

### Protein extraction and Western blots

Total cell lysates were generated in RIPA buffer (PBS with 1% Nonidet P40, 0.5% sodium deoxycholate, 0.1% SDS). Membrane fractions were generated using the Proteoextract Native Membrane kit (Calbiochem), which separates the membrane and membrane-associated proteins, including plasma membrane, endoplasmic reticulum and Golgi membrane proteins, from non-membrane proteins. Proteins were quantitated using the DC assay (Biorad). Westerns used 1-5 μg protein per lane on 7% or 3-8% Tris-Acetate NuPAGE gels with SeeBluePlus2 or HiMark ladders (Invitrogen). Proteins were transferred onto Hybond ECL (GE Healthcare) and detected with antibodies against Ptk7 [21] (1:15000), Celsr1 [71] (1:3000), Scrib [72] (1:500; gift from P. Humbert), β-tubulin (1:5000; Santa Cruz sc-9104), or fatty acid synthase (1:1000; sc-55580) with HRP-conjugated secondary antibodies (1:12,000; DAKO) and detection with ECL Advance (GE Healthcare). Bands were quantitated from exposed films following ECL, using the Geliance 600 Imaging System (Perkin-Elmer). Band intensity was determined using areas of equal size, with background subtraction, and normalised against the values obtained for β-tubulin immunostaining for each lane, to allow for loading differences. Data for the intensity of upper and lower bands were compared across genotypes, using the Upper band value for the wild-type as the reference value (set arbitrarily at 1). Quantitation values are means obtained from the duplicate samples shown in Figure 10G, and the experiment was repeated 3 times with independent samples, yielding similar results.

### Immunofluorescence

Immunofluorescence on 10 μm cryosections was performed on fresh-frozen material; sections were fixed for 10 minutes with either 4% paraformaldehyde or ice-cold methanol, blocked with 10% newborn calf serum and immunostained with antibodies against Scrib (1:200; Santa Cruz sc-28737), Vangl2 (1:50; sc-46561), ZO-2 (1:50; sc-8148), Ptk7 [21] (1:5000), Celsr1 [71] (1:1000) and phalloidin (250 ng/ml; Invitrogen A12381). Cell proliferation rates were determined by staining with antibody against phospho-Histone H3 (1:250 pHH3, Upstate Cell Signaling). Primary antibody staining was detected with appropriate secondary antibodies conjugated with AlexaFluor-488 or AlexaFluor-594 (1:250 dilution; Invitrogen), mounted in Vectashield with DAPI (Vector Laboratories) and imaged with a Zeiss Axiophot microscope. Quantitation of proliferation rates was performed by counting pHH3+ cells and total (DAPI+) cells within the neuroepithelium of E8.5 (4-6 somites) embryos; the mitotic index was calculated as the average from 11 sections from each of three embryos of each genotype, with analysis from the somitic region of the embryo. Immunostaining of the sensory hair cells was performed as described [21].

## Authors' contributions

AP identified the mutation, performed expression analysis, conducted phenotyping studies and participated in writing the manuscript; CD performed Western blotting and participated in writing the manuscript; VP contributed to embryo phenotyping and participated in writing the manuscript; AE performed the ENU screen that identified the chuzhoi mutant; CF supplied Celsr1 antibody and provided advice with Celsr1 analysis; ZL conducted initial genetic mapping studies; SW enabled genetic interaction studies; XL performed the inner ear immunostaining and participated in writing the manuscript; DN coordinated the ENU screen and participated in writing the manuscript; CHD performed the analysis of lung defects and participated in writing the manuscript; DJH performed the analysis of cardiovascular defects and participated in writing the manuscript; JNM conceived of the study, performed initial phenotyping and wrote the manuscript. All authors read and approved the final manuscript.
